# Integrated Genomic Analysis Reveals the Synergistic Role of PNPLA3 and ABCC8 Variants in Diabetic MASLD in Pakistan

**DOI:** 10.3390/medsci13030178

**Published:** 2025-09-05

**Authors:** Asma Shabbir, Ambrina Khatoon, Zaigham Abbas, Sucheta Srivastava, Talat Mirza

**Affiliations:** 1 Department of Pathology, Ziauddin University & Hospital-Clifton, Karachi 75600, Pakistan; asma.12369@zu.edu.pk; 2 Department of Research, Ziauddin University & Hospital-Clifton, Karachi 75600, Pakistan; 3 Department of Gastroenterology & Hepatology, Ziauddin University & Hospital-Clifton, Karachi 75600, Pakistan; drzabbas@gmail.com; 4 Guardant Health, 505 Penobscot Drive, Redwood City, CA 94063, USA; 5 Departments of Research & Molecular Medicine, Ziauddin University & Hospital-Clifton, Karachi 75600, Pakistan; deanresearch@zu.edu.pk

**Keywords:** MASLD, NAFLD, PNPLA3, ABCC8

## Abstract

**Introduction**: Metabolic dysfunction associated steatotic liver disease (MASLD), previously termed as nonalcoholic fatty liver disease (NAFLD), is a growing global health concern, particularly in South Asia. While PNPLA3 is a well-recognized genetic contributor to MASLD, the role of other metabolic genes, such as ABCC8, remains unexplored in South Asian populations. In this study, we aim to investigate the genetic association and potential synergy between PNPLA3 (rs738409) and ABCC8 (rs146378237) variants in MASLD pathogenesis in a Pakistani cohort. **Methods**: A two-phased case–control study was conducted. Whole Exome Sequencing (WES) was performed on 6 MASLD cases and 6 healthy controls to identify relevant variants, followed by validation via Sanger sequencing in an extended MASLD cohort (*n* = 52). Variant frequencies were compared with 96 ethnically matched controls from the 1000 Genomes Project. Furthermore, the association of the variants with clinical, biochemical, and fibrotic parameters was assessed. **Results**: The PNPLA3 rs738409 G allele (MAF = 0.47) and ABCC8 rs146378237 T allele (MAF = 0.36) were significantly enriched in MASLD cases and strongly associated with cirrhosis. The TT genotype of ABCC8 was also linked to T2DM and low HDL levels. Importantly, eight MASLD patients harbored both GG (PNPLA3) and TT (ABCC8) genotype, and all were known cases of diabetes, suggesting a synergistic genetic interaction. **Conclusions**: This is the first report of ABCC8 rs146378237 in a South Asian MASLD cohort, revealing population-specific risk and a gene–gene interaction that may inform targeted screening and personalized management of MASLD in high-risk diabetic individuals.

## 1. Introduction

Non-alcoholic fatty liver disease (NAFLD) has emerged as one of the most common chronic liver disorders worldwide, paralleling the global rise in obesity, type 2 diabetes, and metabolic syndrome [1]. To highlight the centrality of metabolic dysfunction in its pathogenesis, NAFLD has recently been redefined as Metabolic Dysfunction Associated Steatotic Liver Disease (MASLD) [2]. This nomenclature emphasizes the metabolic risk factors that underlie the disease, such as insulin resistance, visceral adiposity, dyslipidemia, and hypertension. Worldwide, MASLD is reported to have a prevalence of 30.05%. Latin America exhibits the highest prevalence of MASLD (44.4%), while Western Europe has the lowest (25.1%) [3]. With the increasing global burden of metabolic disorders, MASLD now represents a significant public health challenge not only in Western countries but also in Asia. South Asian populations appear to be particularly susceptible, as they frequently develop metabolic abnormalities at lower body mass indices—a phenomenon referred to as the “South Asian paradox” [4].

NAFLD/MASLD encompasses a broad spectrum, from benign hepatic steatosis (non-alcoholic fatty liver, NAFL) to non-alcoholic steatohepatitis (NASH), which is characterized by hepatocellular inflammation, ballooning degeneration, and variable degrees of fibrosis. Over time, NASH can progress to advanced fibrosis, cirrhosis, and eventually hepatocellular carcinoma (HCC), which is a major cause of liver-related morbidity and mortality globally and often necessitates liver transplantation [5].

The pathogenesis of MASLD is multifactorial, involving intricate interactions between metabolic, environmental, and genetic influences. Hepatic lipid accumulation resulting from insulin resistance initiates lipotoxicity and oxidative stress, leading to mitochondrial dysfunction and hepatocyte injury. Inflammatory pathways are further activated by dysregulated adipokines and cytokines. Additionally, growing evidence implicates gut dysbiosis and increased intestinal permeability, forming the components of the gut–liver axis in promoting hepatic inflammation and disease progression [6]. These factors collectively contribute to the clinical heterogeneity observed among MASLD patients, including differences in susceptibility, severity, and outcomes.

While lifestyle and metabolic factors are central to MASLD development, genetic predisposition plays a crucial modifying role. Heritability estimates suggest that 27% to 39% of MASLD cases may be attributable to genetic variation [7]. Recent advances in high-throughput genomic technologies, particularly genome-wide association studies (GWAS) and whole-exome sequencing (WES), have greatly enhanced our understanding of the genetic landscape of MASLD. These studies have identified several risk variants, particularly those involved in lipid metabolism, insulin signaling, and inflammatory pathways. Among the most consistently associated genetic variants is rs738409 in the patatin-like phospholipase domain-containing protein 3 (PNPLA3) gene. This single-nucleotide polymorphism (SNP), characterized by a cytosine-to-guanine (C>G) substitution, results in an isoleucine-to-methionine change at position 148 (I148M), impairing the hydrolysis of triglycerides in hepatocytes and promoting intracellular fat accumulation [8]. Numerous studies across diverse ethnicities have confirmed its strong association with increased hepatic fat content, NASH, and fibrosis, making PNPLA3 a robust genetic marker for MASLD progression [8,9]. Another emerging gene of interest is ATP-binding cassette sub-family C member 8 (ABCC8), which encodes the sulfonylurea receptor-1 (SUR1), a component of the ATP-sensitive potassium channel in pancreatic β-cells. This gene plays an essential role in insulin secretion. Variants in ABCC8 have been linked to various disorders of glucose homeostasis, including hyperinsulinemic hypoglycemia and type 2 diabetes [10]. Although ABCC8 has not been as extensively studied in MASLD as PNPLA3, its relevance lies in its potential impact on insulin regulation, a key pathogenic factor in MASLD. Despite these genetic insights, most research has been concentrated in European, East Asian, or Hispanic populations, leaving a substantial gap in our knowledge of ethnic-specific genetic risk factors, particularly in underrepresented regions like South Asia [11].

To address this gap, the present study investigated potential genetic contributors to MASLD in a Pakistani cohort using a next-generation sequencing (NGS)-based whole-exome sequencing (WES) approach. NGS enables high-throughput, base-level resolution of the exome, allowing for the comprehensive detection of both common and rare coding variants that may underlie disease susceptibility. This approach is particularly valuable in genetically diverse and underrepresented populations, where ancestry-specific variants may be missed by studies conducted in predominantly European or East Asian cohorts. Following initial variant prioritization through in silico bioinformatics tools, two key variants were selected for further validation: PNPLA3 rs738409, a well-established MASLD risk variant, and ABCC8 rs146378237, a less characterized but metabolically relevant candidate. By integrating advanced genomic techniques with targeted variant analysis, this study aims to deepen the understanding of the genetic architecture of MASLD in the Pakistani population and contribute to the emerging field of precision hepatogenomics.

## 2. Methodology

### 2.1. Patient Selection

This study was conducted at Ziauddin Hospital and University, Karachi, Pakistan, after obtaining Institutional Review Board (IRB) approval (Reference code: 44101021ASPAT). A total of 58 MASLD cases were recruited from the outpatient Gastroenterology and Hepatology clinic. All patients aged ≥18 years underwent primary screening via ultrasound to diagnose a fatty liver. The exclusion criteria encompassed absence of significant alcohol intake (>30 gm/day for males and >20 g/day for females), pregnant females, individuals with secondary causes of hepatic steatosis or fibrosis such as viral hepatitis, hepatotoxic medications, autoimmune liver diseases, and those with decompensated liver disease. Demographic data, anthropometric measurements, detailed medical history, and laboratory investigations were recorded. Transient elastography was conducted using a Fibrotouch device (HISKY Medical Technologies, Beijing, China). Steatosis severity was assessed via the controlled attenuation parameter (CAP) as follows: S0 (normal: <5% liver fat): up to 240 dB/m, S1 (mild steatosis 5–33%): 241 to 260 dB/m, S2 (moderate steatosis 34–66%): 261 to 290 dB/m, S3 (severe steatosis > 66%): >290 dB/m. Fibrosis staging was categorized as F0–F1: up to 7.4 kPa, F2: 7.5–10 kPa, F3: 10.1–14 kPa, F4: >14 kPa [12,13].

### 2.2. Study Phases

#### 2.2.1. Whole Exome Sequencing (WES) Discovery Cohort

A case–control study design was employed in the discovery phase, comprising 6 MASLD cases selected from the main study pool of 58 MASLD cases and 6 age and gender matched healthy population controls. WES was conducted on these 12 participants (6 cases and 6 controls) to identify candidate genetic variants for downstream validation.

Whole blood samples were collected from all participants (58 cases and 6 controls), and genomic DNA was extracted using the QIAamp DNA Blood Mini Kit (Cat. No. 51104, Qiagen, Hilden, Germany) following the manufacturer’s protocol. DNA concentration and purity were assessed using a Nanodrop spectrophotometer to ensure suitability for sequencing. To identify candidate variants for downstream genotyping, whole-exome sequencing (WES) was performed on a discovery cohort of 12 individuals (6 MASLD cases and 6 healthy population controls). WES was performed by Macrogen Inc. (Seoul, Republic of Korea) using Illumina platform with the Agilent V6 Sure Select XT Low Input Target Enrichment protocol. Raw sequencing reads were converted to FASTQ format using bcl2fastq v2.20.0 and aligned to the human reference genome GRCh38 (hg38) using the Burrows-Wheeler Aligner (BWA v0.7.17). Post-alignment, duplicate reads were removed using Picard tools (v2.19.2), and variant calling was performed using the Genome Analysis Toolkit (GATK v4.0.5.1). Identified variants were annotated using SnpEff (v5.0e). Standard WES QC measures were applied during data processing, including removal of low-confidence variant calls and quality filtering based on base quality and coverage depth. Furthermore, variant classification was performed according to the American College of Medical Genetics guidelines (ACMG) as benign, likely benign, pathogenic (P), likely pathogenic (LP), and variant of uncertain significance (VUS) based on the ClinVar database [14].

Variants identified from WES were subjected to a multi-step filtering process to prioritize potentially pathogenic candidates. Initially, variants were assessed for their predicted functional impact using established in silico tools. Variants with SIFT scores < 0.05, indicating deleterious effects on protein function, and PolyPhen-2 scores equal to or greater than 0.85, predicting probable damaging effects, were retained for further analysis. To ensure relevance to the target population, variants with a minor allele frequency (MAF) greater than 0.05 in South Asian populations—based on publicly available databases such as gnomAD_SAS and the 1000 Genomes Project—were excluded. Additionally, Combined Annotation Dependent Depletion (CADD) scores > 15 were used to further prioritize variants likely to have deleterious effects. Synonymous variants, which do not alter the amino acid sequence and are less likely to affect protein function, were excluded. Finally, duplicate variant calls, which may arise from technical artifacts or repetitive regions, were removed to avoid redundancy and false positives. This rigorous filtering pipeline enabled the selection of candidate variants with a higher likelihood of clinical significance for downstream validation. Among filtered variants, ABCC8 rs146378237 was selected as it was the most commonly observed potentially damaging variant in MASLD cases. Although PNPLA3 was not flagged by these prediction tools, it is a well-characterized functional variant and was therefore included in the analysis based on its established clinical and experimental relevance [15]. These two variants were prioritized for validation in the extended MASLD cohort (*n* = 52) and compared against 96 ethnically matched healthy individuals from the Punjabi population in Pakistan (PJL), obtained from the 1000 Genomes Project [16]

As these PJL cohorts were not phenotypically screened for MASLD, we cautiously interpreted the findings, recognizing that undiagnosed metabolic traits or hepatic steatosis could not be excluded and may impact allele frequency comparisons. Additionally, Hardy–Weinberg equilibrium (HWE) analysis was conducted for both variants in the control group to assess genotyping reliability and population stratification.

#### 2.2.2. Sanger Sequencing (Validation Cohort)

In light of the recent shift in nomenclature from NAFLD to MASLD, this study retrospectively applied the updated terminology while retaining the established clinical stratification used in NAFLD research. A total of 52 MASLD cases were classified into four diagnostic subgroups based on clinical, laboratory, and imaging criteria: non-alcoholic fatty liver (NAFL), non-alcoholic steatohepatitis (NASH), steatofibrosis (SF), and cirrhosis, with 13 patients in each group. NAFL were the cases with steatosis, normal ALT levels, and F0–F1 fibrosis, NASH were grouped with steatosis, raised ALT levels, and F0–F3 fibrosis, SF were grouped with steatosis, normal ALT levels, and F2–F3 fibrosis, and cirrhotic were the cases with steatosis, elevated ALT levels, and F4 fibrosis.

Sanger sequencing was performed on 52 MASLD cases using standard protocols. Primers for rs738409 and rs146378237 were designed using Primer3 software (https://primer3.ut.ee/, accessed on 2 November 2023) with standard parameters and obtained commercially (Macrogen, Peniconpk, 25 nmol). (Table 1) The expected amplicon sizes were 310 bp for rs738409 and 495 bp for rs146378237, based on the reference genome positions flanked by the selected primers. PCR amplification was performed using the DreamTaq PCR kit (Cat. No. K1081, Thermo Fisher Scientific, Waltham, MA, USA). After several optimization attempts at different temperatures, the PCR cycling conditions were as follows: initial denaturation at 94 °C for 5 min followed by 25 cycles of 1 min at 94 °C, 1 min at 50 °C, and 1 min at 72 °C, with final extension of 5 min at 72 °C. PCR products were then analyzed by agarose gel electrophoresis to verify amplification specificity and product size. Following successful amplification, the purified PCR products were subjected to bidirectional Sanger sequencing using the Big Dye Terminator v3.1 Cycle Sequencing Kit (Applied Biosystems, Thermofisher Scientific, Waltham, MA, USA). Sequencing reactions were performed on an ABI 3500 Genetic Analyzer. The resulting chromatograms were analyzed using Sequencing Analysis Software v5.4 and were manually reviewed by two independent reviewers to confirm base calls and ensure sequencing accuracy. Variant annotation was conducted by aligning sequences to the reference genome GRCh38 (hg38) using MEGA X software version 11.

Statistical analysis was conducted using SPSS version 28. Descriptive statistics were used to summarize demographic and clinical characteristics. Continuous variables were expressed as mean ± standard deviation (SD) or median with interquartile range (IQR), and categorical variables as frequencies and percentages. Hardy–Weinberg equilibrium (HWE) was assessed for both variants in the control group. The PNPLA3 rs738409 genotype distribution in WES controls was consistent with HWE (*p* = 0.055, chi-square = 3.672); also, in the PJL cohort, it was within HWE (*p* = 0.823, chi-square = 0.05). For ABCC8 rs146378237, the genotypes were monomorphic (CC genotypes) for both WES controls and the PJL cohort. A formal HWE test could not be performed; however, the distribution does not suggest deviation [17]. Genotype and allele frequencies were compared between cases and controls (PJL) using the Chi-square/Fisher’s exact test. Associations between alleles and genotypes with diagnostic groups, demographic variables, and comorbidities were also performed using the Chi-square/Fisher’s exact test. Cramer’s V was calculated to assess the strength of association with interpretation as follows: values below 0.2 indicate a weak association, 0.2–0.4 a moderate association, and above 0.4 a strong association. Multiple linear regression was used to examine the associations between genotypes and clinical parameters. A *p*-value < 0.05 was considered statistically significant. Graphs and visualizations were generated using GraphPad Prism version 10.4.1, SR Plot [18], and Biorender [19].

## 3. Results

### 3.1. Baseline Characteristics and Analysis of the Discovery Cohort

The discovery cohort included 12 participants: 6 MASLD cases and 6 healthy controls matched with age and gender. All MASLD cases had T2DM; additionally, three were hypertensive and three had dyslipidemia. Among the MASLD cases, five had steatosis grade S3 and one had grade S2. Fibrosis staging revealed two cases each with F0–F1, F2, and F4 fibrosis (Supplementary Figure S1).

A preliminary ClinVar-based classification of WES variants identified in the discovery cohort was performed, revealing multiple pathogenic, likely pathogenic, and VUS variants. The variant distribution is visualized as a heatmap in Supplementary Figure S2, providing an overview of ClinVar annotation categories across all identified variants.

After applying prediction tools including SIFT, PolyPhen-2, gnomAD_SAS, and CADD, the ABCC8 rs146378237 variant was identified in 50% of MASLD cases. In contrast, the PNPLA3 rs738409 variant was observed in 66.6% of MASLD cases, based on direct genotyping data without in silico prediction filters.

### 3.2. Baseline Characteristics of the Validation Cohort

The validation cohort included 52 MASLD cases, evenly divided into four diagnostic subgroups: NAFL, NASH, steatofibrosis (SF), and cirrhosis, with 13 patients in each group. The majority of patients were male (*n* = 36, 69.2%), while 16 (30.8%) were female (Table 2).

### 3.3. Association of rs738409 and rs146378237 Polymorphisms with Disease Susceptibility

**Figure 1:**
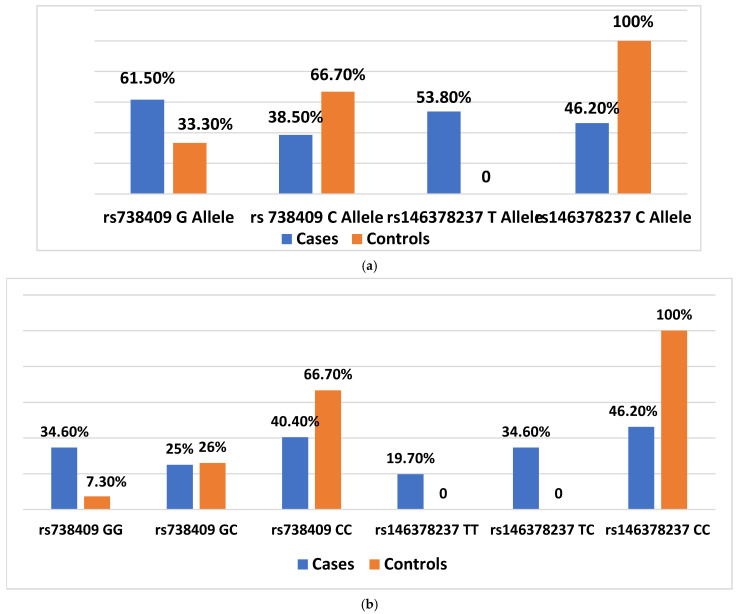
(**a**) Frequency of alleles; (**b**) Frequency of genotype. Controls: Punjabi population in Pakistan (PJL) from the 1000 Genomes Project.

Analysis showed a significant association between both rs738409 and rs146378237 variants and disease status. For rs738409, the minor allele frequency (MAF) of the G (risk) allele (MAF = 0.47) was more frequent in cases (*n* = 32/52, 61.5%) compared to PJL (*n* = 32/96, 33.3%) (MAF = 0.20), with a significant *p*-value (*p* = 0.001). The GG genotype was also more prevalent among cases (*n* = 18/52, 34.6%) than in PJL (*n* = 7/96, 7.3%). Similarly, the T (risk) allele of rs146378237 was observed more frequently in cases (*n* = 28/52, 53.8%) (MAF = 0.36), whereas it was absent in PJL (MAF = 0), and the homozygous TT genotype appeared more commonly in the diseased group (*n* = 10/52, 19.7%). In contrast, the reference alleles and their respective genotypes for both the variants were predominantly found in the PJL reference cohort. As the PJL group represents an unscreened general population sample, these associations serve primarily as hypothesis-generating and indicate a potential genetic contribution of both variants to disease susceptibility. (Figure 1).

### 3.4. Distribution of Alleles and Genotypes Across Disease Subtypes

Analysis revealed a statistically significant association between both rs738409 and rs146378237 variants and disease subtypes. For rs738409, the G allele (92.3%) and GG genotype (69.2%) were significantly more frequent in cirrhosis cases as compared to other groups (*p* = 0.021), (*p* = 0.008), respectively. In contrast, the reference C allele and CC genotype were more frequent in NAFL and NASH. Similarly, for rs146378237, the T allele (92.3%) and TT genotype (46.2%) were significantly more common in cirrhosis (*p* = 0.008), (*p* = 0.023), respectively.

**Figure 2:**
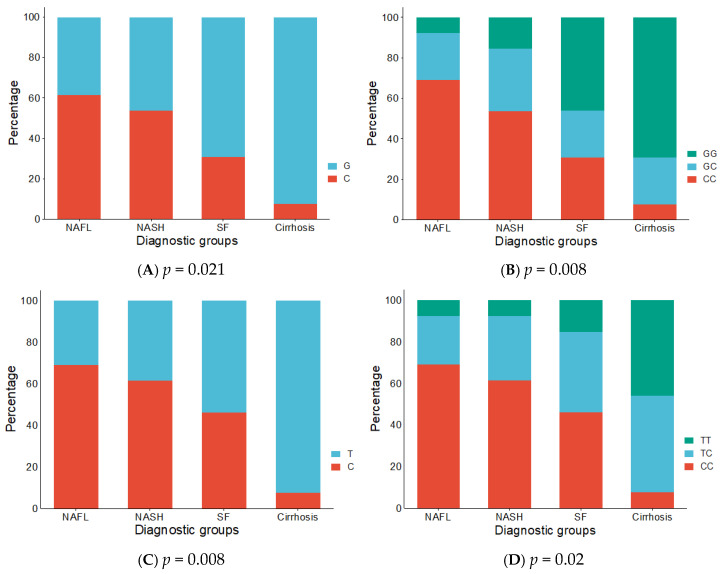
The proportions of cases with alleles and genotypes of PNPLA3 rs738409 C>G and ABCC8 rs416378237 C>T in various diagnostic groups. (**A**) The proportions of cases with alleles of PNPLA3 rs738409 C>G in diagnostic groups. (**B**) The proportions of cases with genotypes of PNPLA3 rs738409 C>G in diagnostic groups. (**C**) The proportions of cases with alleles of ABCC8 rs416378237 C>T in diagnostic groups. (**D**) The proportions of cases with genotypes of ABCC8 rs416378237 C>T in diagnostic groups. *p*-value estimated by Chi-Square or Fisher’s Exact tests for categorical datasets.

Overall, the minor/risk alleles (G in rs738409 and T in rs146378237) and their respective homozygous genotypes (GG and TT) were significantly more prevalent in patients with advanced liver disease stages, particularly cirrhosis. Conversely, the reference alleles and genotypes (C and CC) were more commonly observed in milder disease stages such as NAFL and NASH. (Figure 2, Supplementary Table S1).

### 3.5. Analysis of rs738409 and rs146378237 Variants in Relation to Demographics and Comorbidities

The analysis revealed no statistically significant associations between the variants rs738409 and rs146378237 and demographic variables, such as age, gender, and ethnicity (Supplementary Table S2). However, a significant association was observed between rs146378237 and diabetes mellitus, with the risk allele T present in 67.9% of diabetic individuals (*p* = 0.029) and the risk genotype TT found in 35.7% of diabetics (*p* = 0.004) (Table 3).

### 3.6. Potential Synergistic Effect of rs738409 and rs146378237 in MASLD Cases

**Figure 3:**
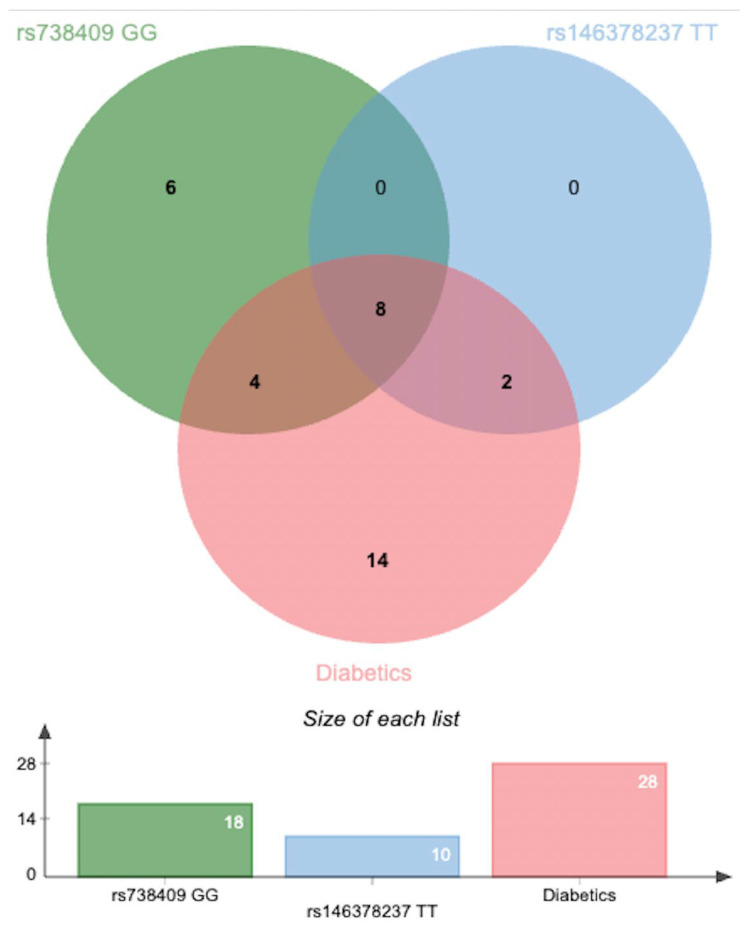
Venn diagram illustrating the overlap between the TT genotype of rs146378237, the GG genotype of rs738409, and diabetes mellitus in the MASLD cohort (*n* = 52). A total of 8 patients carried both risk genotypes and had diabetes, suggesting a potential synergistic effect [20].

To assess potential interactions between genetic risk factors, the co-occurrence of rs738409 (GG genotype) and rs146378237 (TT genotype) was examined across the MASLD cohort. Among the 52 MASLD patients, 18 individuals carried the GG genotype of rs738409, and 10 carried the TT genotype of rs146378237. Notably, 8 patients were found to harbor both risk genotypes simultaneously. All 8 of these individuals were also diagnosed with T2DM, suggesting a possible synergistic or additive genetic effect specifically among diabetic patients. While this co-occurrence is noteworthy, it should be interpreted as a preliminary observation, suggesting further studies to confirm this association in larger cohorts. (Figure 3) (Supplementary Figure S3).

To further elucidate the effects of the genotypes with clinical and biochemical covariates, multiple linear regression analysis was performed. Regression analysis showed significant associations of the rs738409 (GG and GC genotypes) and rs146378237 (TC genotype) with key clinical and biochemical parameters. Notably, NAFLD cases carrying the rs738409 GG genotype showed a significant increase in ALT levels by 26.994 units (*p* = 0.002) and fibrosis scores by 3.648 units (*p* = 0.047). In addition, the rs146378237 TC genotype was significantly associated with reduced HDL levels by 9.297 (*p* = 0.014) (Supplementary Table S3).

## 4. Discussion

MASLD is a multifactorial disorder arising from complex interactions between genetic and environmental factors. In Pakistan, the estimated pooled prevalence of MASLD in the general population is 29.02%, with a significantly higher prevalence of 58.7% among individuals with diabetes [21]. This study is the first of its kind in the Pakistani population to utilize whole exome sequencing to identify SNPs associated with MASLD, validate them through Sanger sequencing, and investigate their associations with biochemical parameters in MASLD cases.

In the present study, the minor allele frequency (MAF) of the PNPLA3 rs738409 G allele was 0.47 among MASLD patients. This frequency is notably higher than the global averages reported in healthy South Asian populations in the 1000 Genomes and gnomAD databases (MAF ~0.35–0.38) and considerably elevated compared to the healthy Punjabi Pakistani population (MAF ~0.20), suggesting a strong enrichment of the G allele in MASLD cases [16,22]. Our findings align closely with the founding Dallas Heart Study by Romeo et al. (2008), which reported a G allele MAF of 0.49 in Hispanic individuals, where the GG genotype was associated with a two-fold increase in liver fat content measured by Magnetic Resonance Imaging (MRI) [23]. Lower MAFs and GG genotype frequencies were reported in European Americans (0.23) and African Americans (0.17), highlighting ethnic variability and a strong association between the GG genotype and hepatic steatosis [23]. Furthermore, South Asian studies—particularly those from South India—have reported PNPLA3 G allele MAFs ranging from 0.38 to 0.40, consistent with our findings [24,25,26]. A study by Mausumi Das et al. from West Bengal reported a similar MAF of 0.37 in MASLD cases, further supporting the variant’s relevance in Eastern South Asian populations [27]. Additionally, a multi-ethnic South Asian study by Zain et al. reported a comparable G allele frequency (~0.40) across diverse South Asian ethnicities, including Indian subgroups, reinforcing the potential contribution of this variant to MASLD risk across the region [28]. Complementing these findings, population-specific data from the 1000 Genomes South Asian subgroups show G allele frequencies of 0.24 in BEB (Bengali in Bangladesh), 0.311 in GIH (Gujarati Indian in Houston), 0.22 in ITU (Indian Telugu in the UK), and 0.25 in STU (Sri Lankan Tamil in the UK) [16]. These consistently moderate-to-high frequencies suggest that the PNPLA3 G allele is broadly prevalent across South Asian subpopulations and may play a significant role in disease susceptibility.

In our study, the GG genotype was more frequent among MASLD cases (34.6%), while the CC genotype predominated in healthy populations from the Punjabi Pakistani (PJL) cohort (66.7%), consistent with the previous literature reporting a risk genotype (GG) higher in MASLD cases and the studies also reported a GC genotype higher in the cases as compared to controls [24,29,30]. However, in our study, the GC genotype showed similar proportions in cases (25%) and the PJL cohort (26%). Our controls were derived from the Punjabi Pakistani cohort of the 1000 Genomes Project, representing a general population sample without documented liver disease. The discrepancy in GC genotype distribution might be explained by differences in control group characterization or potential undiagnosed metabolic conditions within the reference population. While the PJL cohort was not phenotypically screened for MASLD, its use as an ethnically matched genetic reference is consistent with standard practice in genomic association studies. As no disease outcomes were attributed to the PJL group, allele frequency comparisons were interpreted appropriately, without assuming clinical classification. Additionally, environmental and lifestyle factors not captured in public databases might influence genotype–phenotype relationships. However, our study observed GG+GC as a risk factor for MASLD cases in our population, which suggests an enrichment of the risk genotype among affected individuals. Notably, 33.3% of individuals from the PJL cohort in the 1000 Genomes Project carry the GG risk genotype of PNPLA3 rs738409, indicating a substantial background prevalence of this pathogenic variant [16]. This observation may reflect a considerable burden of undiagnosed MASLD in the general PJL population and underscores the potential utility of public genome datasets in identifying populations at elevated genetic risk.

A particularly novel and clinically significant finding was the identification of the ABCC8 rs146378237 variant with MAF of 0.36 among MASLD patients (TT: 19.7%, TC: 34.6%, CC: 46.2%). The rs146378237 variant in the ABCC8 gene involves a C>T substitution at the genomic level (Chr11:17428371C>T, GRCh38), corresponding to c.1958G>A in the canonical transcript NM_000352.6, and results in an amino acid change p.Arg653Gln (R653Q) in the SUR1 protein [31]. Structurally, this mutation lies within the ATP-binding cassette domain, which is critical for ATP sensing and channel gating. Arginine is positively charged and facilitates salt-bridge formation and electrostatic interactions essential for conformational stability. Its substitution by a polar but uncharged glutamine may disrupt intramolecular interactions, affecting channel function and insulin release. This allele is extremely rare in global populations, with a frequency of only 0.00006 reported in the Genome Aggregation Database (gnomAD), underscoring its potential population specificity [21]. Notably, the absence of the T allele has been reported in the PJL cohort from the 1000 Genomes Project, reinforcing the importance of investigating diverse ethnic cohorts in MASLD-related genetic studies. The difference in allele frequency provides a strong preliminary signal suggestive of population-specific enrichment and warrants further investigation in clinically characterized cohorts.

Interestingly, a recent study detected ABCC8 variants in 14.5% of obese MASLD patients, with no mutations found in non-obese MASLD patients [32]. This study utilized digital PCR in peripheral blood mononuclear cell (PBMC) samples and reported a potential link between ABCC8 mutations and progression toward liver cancer. This finding is consistent with our observation of a substantially higher frequency of the T allele (50%) in the discovery cohort and (36%) in our validation cohort and supports a possible role of ABCC8 variants in obesity-associated MASLD pathogenesis. Such evidence highlights the clinical relevance of ethnic and obesity specific genetic screening, particularly in underrepresented populations.

We found significant associations of the PNPLA3 rs738409 G allele (92.3%) and GG genotype (69.2%) with cirrhosis among MASLD patients (*p* = 0.021 and *p* = 0.008, respectively), reinforcing the established role of PNPLA3 in hepatic steatosis and fibrosis progression [9,33]. Our findings also align with the existing literature, as we observed a significant association between the PNPLA3 risk genotypes (GG and GC) and elevated serum ALT levels, indicative of increased hepatocellular injury in genetically susceptible individuals. This observation supports the growing body of evidence linking PNPLA3 polymorphisms with liver injury markers in MASLD. However, our results stand in contrast to those reported by Altaf et al. in a Pakistani cohort, where they observed an association of risk genotype with moderate fatty liver and found no significant association between PNPLA3 variants and biochemical parameters [30]. This discrepancy might be attributed to methodological differences, particularly the use of ultrasonography in their study for assessing hepatic pathology. Ultrasonography, while widely accessible, lacks the sensitivity and specificity required for accurate staging of hepatic fibrosis, potentially limiting the detection of subtle genotype–phenotype correlations. In contrast, our use of transient elastography provided a more precise non-invasive assessment of hepatic fibrosis, enhancing the reliability of our phenotypic classifications. Furthermore, the genetic associations observed in our study reinforce the clinical utility of the ALT-FibroScan diagnostic algorithm we previously proposed and validated in our population [34]. This integrated approach, combining biochemical and imaging markers to improve the non-invasive stratification of MASLD subtypes, offers a practical framework for risk prediction and personalized disease management in resource-limited settings. Moreover, the therapeutic implications of PNPLA3 genotyping are increasingly recognized. Experimental models using patient-derived liver spheroids have demonstrated heightened steatosis and fibrogenesis in PNPLA3 GG carriers, with differential responses to emerging therapies such as resmetirom, a thyroid hormone receptor-β agonist [35]. These observations underscore the potential for genotype-informed personalized treatment strategies.

Similarly, ABCC8 rs146378237 demonstrated a significant enrichment in cirrhotic MASLD patients (T allele frequency 92.3%, TT genotype 46.2%; *p* = 0.008 and *p* = 0.023, respectively) and was associated with reduced HDL cholesterol levels (*p* = 0.014). The association of ABCC8 with low HDL in our study might be attributed to its role in insulin secretion and glucose metabolism, which indirectly influences lipid homeostasis. Dysfunctional variants may impair insulin release, contributing to insulin resistance, which in turn affects HDL synthesis and reverse cholesterol transport. This supports a multifactorial role for ABCC8 genetic variation in metabolic and hepatic disease, particularly within our South Asian Pakistani cohort. Notably, while the PNPLA3 rs738409 variant is consistently implicated in hepatic lipid accumulation and fibrosis, its association with systemic metabolic syndrome features such as insulin resistance and T2DM remains inconsistent, a pattern mirrored in our findings [35,36,37]. Conversely, the ABCC8 rs146378237 demonstrated a robust association with T2DM in our cohort. This finding aligns with recent genetic studies across diverse populations. For instance, a study in a Kinh Vietnamese population identified significant associations between ABCC8 single-nucleotide polymorphisms and T2DM susceptibility [38].

In our validation cohort, we identified a distinctive subgroup of eight MASLD patients co-harboring two pathogenic variants: PNPLA3 rs738409 (I148M) and ABCC8 rs146378237 (R653Q), all of whom were also diagnosed with T2DM. This dual-variant occurrence suggests a potential molecular interaction contributing to disease severity, particularly in the diabetic MASLD subset. The ABCC8 gene is known to harbor ~ 14 activating variants linked to neonatal diabetes mellitus (NDM) and over 300 inactivating mutations associated with congenital hyperinsulinism via their impact on the SUR1-regulated K_ATP_ channel [39]. While the functional effect of R653Q has not yet been biochemically validated, it lies within a highly conserved domain of nucleotide-binding domain 1 (NBD1) of SUR_1_ [40]. Multiple in silico prediction tools—including SIFT, PolyPhen-2, CADD, and ProtVar—indicate it is deleterious, impairing ATP-mediated channel closure and thereby reducing insulin secretion [41]. Functionally, impaired insulin release driven by ABCC8 R653Q may initiate chronic postprandial hyperglycemia, which contributes to insulin resistance through downregulation of IRS1 and the PI3K–Akt pathway [40,42]. Concurrently, AMPK suppression and ChREBP/mTORC1 activation promote de novo lipogenesis, exacerbating hepatic steatosis and inflammation [43,44]. Inactivating mutations result in hyperinsulinemia and upregulate the IRS1 and PI3K-Akt pathway, causing increased lipogenesis [43,45]. This metabolic imbalance is further intensified by PNPLA3 I148M, a well-established genetic determinant of hepatic fat accumulation, which disrupts triglyceride hydrolysis and promotes lipid retention in hepatocytes. The co-occurrence of PNPLA3 and ABCC8 risk alleles represents a dual pathogenic hit, in which hepatic lipid metabolism is compromised by PNPLA3, while β-cell dysfunction and impaired insulin dynamics are driven by ABCC8. This gene–gene interaction accelerates disease progression through lipotoxicity, glucotoxicity, chronic inflammation, and fibrogenesis. Notably, this is the first report to our knowledge of the ABCC8 R653Q variant co-segregating with PNPLA3 I148M in MASLD patients, highlighting the novelty of our findings and supporting the rationale for integrated genomic and metabolic risk profiling in MASLD, particularly in patients with diabetes. However, the cross-sectional design of the study limits assessment of temporality or causality between genotype, diabetes onset, and MASLD progression. Validation in larger, longitudinal cohorts is needed to further elucidate the association.

A key strength of this study is its pioneering application of WES in a Pakistani MASLD cohort, followed by validation through Sanger sequencing. This dual approach enabled the identification of a well-established MASLD-associated variant (PNPLA3 rs738409) and the discovery of a rare, population-specific variant (ABCC8 rs146378237). The integration of genomic data with clinical, biochemical, and fibrotic markers added depth and translational value, while the identification of a potential gene–gene interaction in diabetic MASLD patients enhanced the novelty of the findings. Nevertheless, the small sample size in the discovery phase may affect the sensitivity of variant detection and reduce the reliability of identifying rare variants. However, this limitation is common to exploratory WES studies, particularly in underrepresented populations, and our study followed the same approach. Importantly, this was addressed by validating prioritized variants in a larger cohort, thereby enhancing confidence in the observed associations. However, to substantiate these findings, larger, multicenter studies across diverse South Asian populations are warranted. Additionally, functional investigations into the ABCC8 variant are essential to better understand its mechanistic role in hepatic and metabolic dysfunction. While the presence of the ABCC8 R653Q variant was confirmed through Sanger sequencing, its functional impact was inferred using in silico prediction tools. Future in vitro and in vivo studies are needed to experimentally validate its biological consequences. Furthermore, while subgroup stratification in the validation cohort allowed preliminary comparisons across different stages of MASLD, the limited number of patients in each category—particularly those harboring both PNPLA3 and ABCC8 risk variants—may restrict the strength of interaction analyses. However, future studies with larger cohorts are necessary to functionally characterize the ABCC8 variant and confirm these interactions. Beyond genetic association, the identification of variants such as PNPLA3 rs738409 and ABCC8 R653Q in this cohort not only reinforces their association with MASLD but also opens avenues for clinical translation. The co-occurrence of these variants, particularly in diabetic MASLD patients, suggests a potential role for genetic screening in identifying individuals at higher risk for advanced liver involvement. In clinical settings, such markers could contribute to early detection strategies, risk stratification, and possibly inform more targeted lifestyle or pharmacological interventions in predisposed individuals. This may be especially relevant in South Asian populations, where the burden of metabolic diseases is high, yet genetic screening remains underutilized.

## 5. Conclusions

In conclusion, this study presents an integrated genomic analysis that reveals a distinct MASLD-associated genetic profile in a Pakistani cohort, wherein the co-occurrence of PNPLA3 rs738409 and ABCC8 rs146378237 identifies a subgroup potentially at higher risk for synergistic metabolic dysfunction. These preliminary findings suggest the hypothesis that dual genetic risk may contribute to disease severity, particularly in diabetic individuals. However, this integrated approach may guide future studies toward the development of population-specific genomic tools for risk stratification and personalized MASLD management.

## Figures and Tables

**Table 1:** Primer sequences for PNPLA3 rs738409 and ABCC8 rs146378237.

Gene	SNP ID	Primer Sequence	Length (bp)	Tm	GC%
**PNPLA3**	**rs738409**				
	Forward Primer	GCATTTTCAAGTTTGTTGCCCTG	23	60	43
	Reverse Primer	CTGAAAGGCAGTGAGGCATGG	21	61	57
**ABCC8**	**rs146378237**				
	Forward Primer	GCATGCAGCTTTCTGGCTTTC	21	61	52
	Reverse Primer	TGAGGGGTGTCTCTGTGCTTC	21	61	57

SNP: Single nucleotide polymorphism; bp: base pair; Tm: Melting temperature.

**Table 2:** Baseline demographic and biochemical characteristics of MASLD cases (*n* = 52).

Characteristics	Total (*n* = 52) Median [IQR]/Mean ± SD
Age	49.46 ± 13.64
Weight	80 [21]
Height	165.23 ± 9.95
BMI	28.65 [9.2]
ALT	27.5 [37]
AST	34.5 [21]
ALP	78 [55.38]
GGT	35.5 [49]
Total Bilirubin	0.6 [0.48]
Direct Bilirubin	0.2 [0.17]
Albumin	3.89 ± 0.79
Platelet count	253 [133]
HbA1c	6.03 ± 1.02
FBS	96 [26]
Serum Insulin	14.31 [7.38]
HOMA-IR	3.50 [2.22]
Total Cholesterol	158.48 ± 37.35
Triglycerides	140 [70]
HDL	42 [13]
LDL	108.15 ± 34.77
Steatosis score	296.38 ± 28.94
Fibrosis score	9.05 [8.1]

BMI: body mass index; ALT: alanine aminotransferase; AST: Aspartate transaminase; ALP: Alkaline phosphatase; GGT: gamma-glutamyl transpeptidase; FBS: fasting blood sugar; TG: Triglyceride; HDL: high-density lipoprotein; LDL: low-density lipoprotein.

**Table 3:** Association between rs738409 and rs46378237 with comorbidities.

Variables	Variants									
	rs738409 Allele		rs738409 Genotype			rs146378237 Allele		rs146378237 Genotype		
	C (Ref.)	G (Alt.)	CC (Ref.)	GC (Alt.)	GG (Alt.)	C (Ref.)	T (Alt.)	CC (Ref.)	TC (Alt.)	TT (Alt.)
**HTN *n* (%)**										
**Present**	13 (40.6)	19 (59.4)	13 (40.6)	7 (21.9)	12 (37.5)	13 (40.6)	19 (59.4)	13 (40.6)	13 (40.6)	6 (18.8)
**Absent**	7 (35)	13 (65)	8 (40)	6 (30)	6 (30)	11 (55)	9 (45)	11 (55)	5 (25)	4 (20)
	*p* = 0.685 ^a^ 0.056 ^b^		*p* = 0.769 ^a^ 0.101 ^b^			*p* = 0.312 ^a^ 0.140 ^b^		*p* = 0.489 ^a^ 0.166 ^b^		
**DM *n* (%)**										
**Present**	12 (42.9)	16 (57.1)	12 (42.9)	4 (14.3)	12 (42.9)	9 (32.1)	19 (67.9)	9 (32.1)	9 (32.1)	10 (35.7)
**Absent**	8 (33.3)	16 (66.7)	9 (37.5)	9 (37.5)	6 (25)	15 (62.5)	9 (37.5)	15 (62.5)	9 (37.5)	0
	*p* = 0.482 ^a^ 0.098 ^b^		*p* = 0.131 ^a^ 0.280 ^b^			*p* = 0.029 ^a,^* 0.304 ^b^		*p* = 0.004 ^a,^* 0.465 ^b^		
**Dyslipidemia *n* (%)**										
**Present**	10 (32.3)	21 (67.7)	11 (35.5)	8 (25.8)	12 (38.7)	14 (45.2)	17 (54.8)	14 (45.2)	11 (35.5)	6 (19.4)
**Absent**	10 (47.6)	11 (52.4)	10 (47.6)	5 (23.8)	6 (28.6)	10 (47.6)	11 (52.4)	10 (47.6)	7 (33.3)	4 (19)
	*p* = 0.264 ^a^ 0.155 ^b^		*p* = 0.654 ^a^ 0.128 ^b^			*p* = 0.862 ^a^ 0.024 ^b^		*p* = 0.983 ^a^ 0.025 ^b^		

^a^ Chi-square test applied. HTN: Hypertension; DM: Diabetes Mellitus; Ref.: Reference; Alt.: Altered. * Significant at *p* < 0.05; ^b^ Cramer’s V value.

## Data Availability

The original contributions presented in this study are included in the article/Supplementary Materials. Further inquiries can be directed to the corresponding author.

## Supplementary Materials

The following supporting information can be downloaded at https://www.mdpi.com/article/10.3390/medsci13030178/s1: Figure S1: Descriptive comparison of baseline clinical parameters between MASLD cases (A) and healthy controls (B). Figure S2: Heatmap displaying variants identified by CLINVAR analysis. Figure S3: Integrated effects of ABCC8 and PNPLA3 variants on hepatic insulin signaling and lipid metabolism. Table S1: Association of variants with NAFLD severity. Table S2: Association of variants with demographic variables. Table S3: Multiple linear regression of lab parameters with rs738409 and rs146378237.

## Abbreviations

The following abbreviations are used in this manuscript:

NAFLD	Non-alcoholic fatty liver disease
NAFL	Non-alcoholic fatty liver
SF	Steatofibrosis
NASH	Non-alcoholic steatohepatitis
MASLD	Metaboloic dysfunction associated steatotic liver disease
BMI	body mass index
ALT	alanine aminotransferase
AST	Aspartate transaminase
ALP	Alkaline phosphatase
GGT	gamma-glutamyl transpeptidase
FBS	fasting blood sugar
TG	Triglyceride
HDL	High density lipoprotein
LDL	Low density lipoprotein
