# Supplementary material for: Integrated Genomic Analysis Reveals the Synergistic Role of PNPLA3 and ABCC8 Variants in Diabetic MASLD in Pakistan
Source: Med Sci (Basel). 2025 Sep 5;13(3):178. doi: 10.3390/medsci13030178 (PMC12452525; doi:10.3390/medsci13030178)
Supplement: Supplementary file 1 [file medsci-13-00178-s001.zip › Figure S1.pdf]

Supplementary Figures

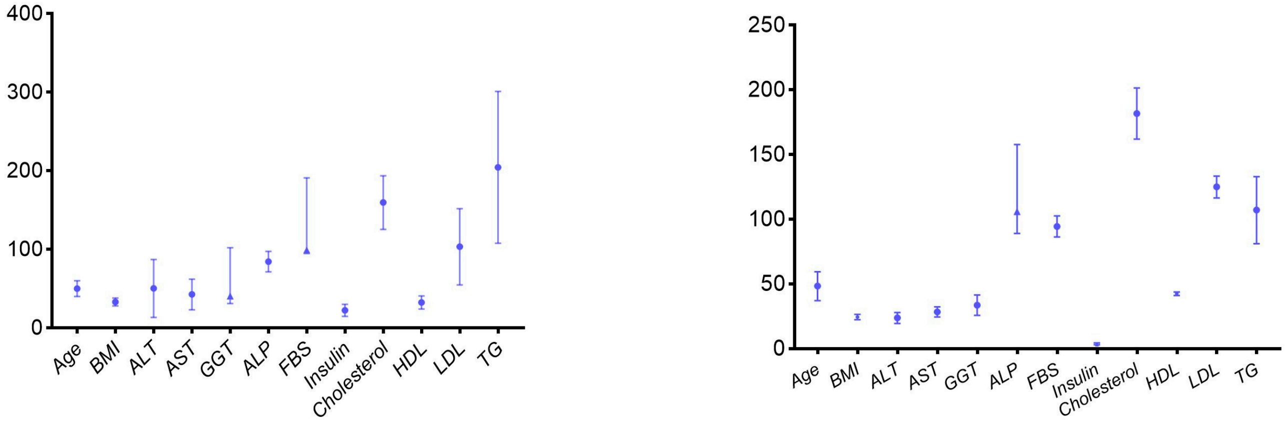

(A) MASLD Cases **(B) Healthy controls**

Figure S1: Descriptive comparison of baseline clinical parameters between MASLD cases (A) and healthy controls (B).

The figure illustrates mean ± standard deviation (•) and median with interquartile range (▲) for key anthropometric and biochemical variables.

BMI: body mass index; ALT: alanine aminotransferase; AST: Aspartate transaminase; ALP: Alkaline phosphatase; GGT: gamma-glutamyl transpeptidase; FBS: fasting blood sugar; TG: Triglyceride; HDL: High density lipoprotein; LDL: Low density lipoprotein
