# Supplementary material for: Integrated Genomic Analysis Reveals the Synergistic Role of PNPLA3 and ABCC8 Variants in Diabetic MASLD in Pakistan
Source: Med Sci (Basel). 2025 Sep 5;13(3):178. doi: 10.3390/medsci13030178 (PMC12452525; doi:10.3390/medsci13030178)
Supplement: Supplementary file 1 [file medsci-13-00178-s001.zip › Figure S3.pdf]

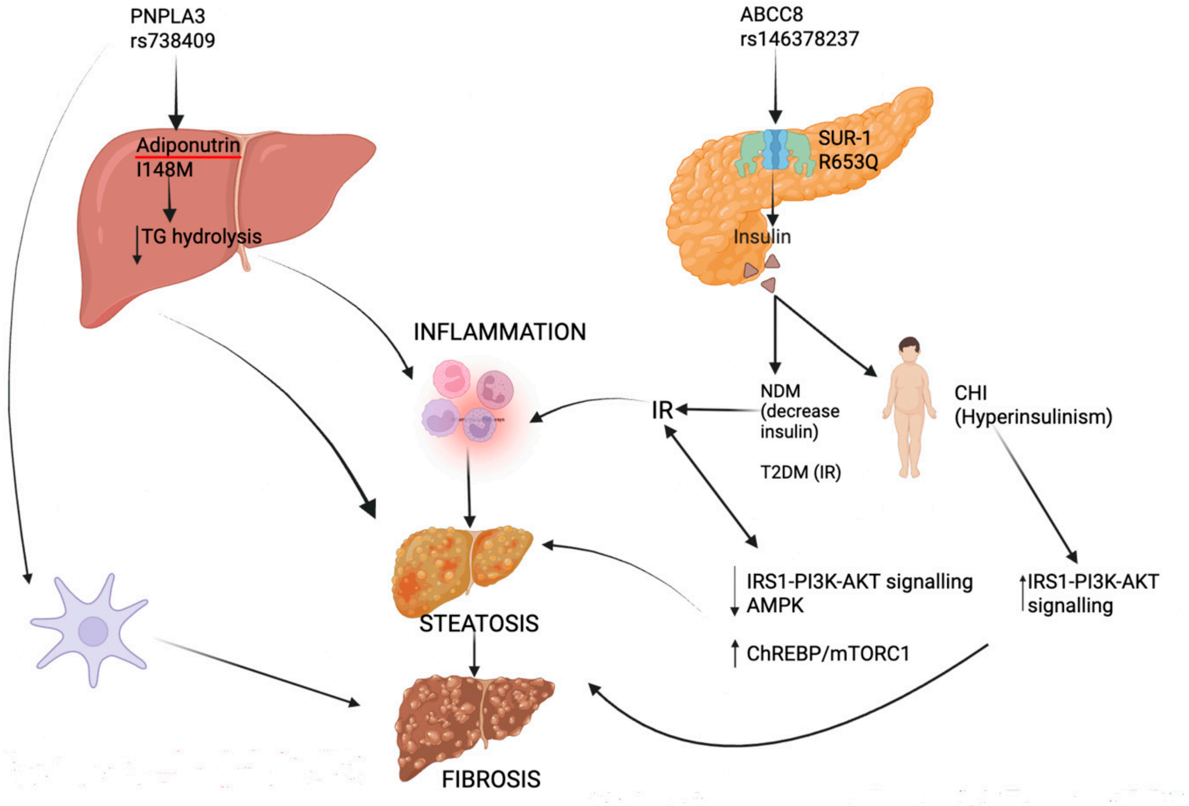

Figure S3: Integrated effects of ABCC8 and PNPLA3 variants on hepatic insulin signaling and lipid metabolism.

The ABCC8 R653Q variant alters insulin dynamics, resulting in either hypoinsulinemia (as in NDM, T2DM) or hyperinsulinemia (as in CHI), both of which impair hepatic insulin signaling via IRS1–PI3K–AKT, AMPK, and mTORC1 pathways. These disruptions enhance gluconeogenesis and lipogenesis. Separately, PNPLA3 I148M impairs hepatic triglyceride hydrolysis, leading to intracellular lipid accumulation. The co-occurrence of these variants in the same individual intensifies hepatic metabolic dysfunction through complementary and compounding mechanisms, exacerbating steatosis, inflammation, and accelerating progression to fibrosis.

NDM: Neonatal Diabetes Mellitus, T2DM: Type-2 Diabetes Mellitus, CHI: Congenital Hyperinsulinism
