# Supplementary material for: Integrated Genomic Analysis Reveals the Synergistic Role of PNPLA3 and ABCC8 Variants in Diabetic MASLD in Pakistan
Source: Med Sci (Basel). 2025 Sep 5;13(3):178. doi: 10.3390/medsci13030178 (PMC12452525; doi:10.3390/medsci13030178)
Supplement: Supplementary file 1 [file medsci-13-00178-s001.zip › Table S1.pdf]

Table S1: Association of variants with NAFLD severity

Variables	Variants									
	rs738409 allele		rs738409 genotype			rs146378237 allele		rs146378237 genotype		
	C (Ref.)	G (Alt.)	CC (Ref.)	GC (Alt.)	GG (Alt.)	C (Ref.)	T (Alt.)	CC (Ref.)	TC (Alt.)	TT (Alt.)
NAFL n (%)	8 (61.5%)	5 (38.5)	9 (69.2)	3 (23.1)	1 (7.7)	9 (69.2)	4 (30.8)	9 (69.2)	3 (23.1)	1 (7.7)
NASH n (%)	7 (53.8)	6 (46.2)	7 (53.8)	4 (30.8)	2 (15.4)	8 (61.5)	5 (38.5)	8 (61.5)	4 (30.8)	1 (7.7)
SF n (%)	4 (30.8)	9 (69.2)	4 (30.8)	3 (23.1)	6 (46.2)	6 (46.2)	7 (53.8)	6 (46.2)	5 (38.5)	2 (15.4)
Cirrhosis n (%)	1 (7.7)	12 (92.3)	1 (7.7)	3 (23.1)	9 (69.2)	1 (7.7)	12 (92.3)	1 (7.7)	6 (46.2)	6 (46.2)
	p = 0.021[a*] 0.433[c]		p = 0.008[b*] 0.396[c]			p = 0.008[a*] 0.476[c]		p = 0.023[b*] 0.370[c]		

[a] Chi square test applied
[b] Fishers exact test applied
*Significant at $p < 0.05$

[c] Cramer's V value

NAFL: Nonalcoholic fatty liver; NASH: Nonalcoholic steatohepatitis; SF: Steatofibrosis; Ref.: Reference; Alt.: Altered
