# Supplementary material for: Integrated Genomic Analysis Reveals the Synergistic Role of PNPLA3 and ABCC8 Variants in Diabetic MASLD in Pakistan
Source: Med Sci (Basel). 2025 Sep 5;13(3):178. doi: 10.3390/medsci13030178 (PMC12452525; doi:10.3390/medsci13030178)
Supplement: Supplementary file 1 [file medsci-13-00178-s001.zip › Table S2.pdf]

## Table S2: Association of variants with demographic variables

| Variables | Variants | | | | | | | | | |
|---|---|---|---|---|---|---|---|---|---|---|
| | rs738409 allele | | rs738409 genotype | | | rs146378237 allele | | rs146378237 genotype | | |
| | C (Ref.) | G (Alt.) | CC (Ref.) | GC (Alt.) | GG (Alt.) | C (Ref.) | T (Alt.) | CC (Ref.) | TC (Alt.) | TT (Alt.) |
| **Age (years) n (%)** | | | | | | | | | | |
| **21-35** | 3 (27.3) | 8 (72.7) | 3 (27.3) | 4 (36.4) | 4 (36.4) | 3 (27.3) | 8 (72.7) | 3 (27.3) | 6 (54.5) | 2 (18.2) |
| **36-50** | 7 (38.9) | 11 (61.1) | 8 (44.4) | 5 (27.8) | 5 (27.8) | 10 (55.6) | 8 (44.4) | 10 (55.6) | 5 (27.8) | 3 (16.7) |
| **51-65** | 6 (40) | 9 (60) | 6 (40) | 4 (26.7) | 5 (33.3) | 9 (60) | 6 (40) | 9 (60) | 3 (20) | 3 (20) |
| **>65** | 4 (50) | 4 (50) | 4 (50) | 0 | 4 (50) | 2 (25) | 6 (75) | 2 (25) | 4 (50) | 2 (25) |
| | p = 0.788[b] 0.142[c] | | p = 0.631[b] 0.200[c] | | | p = 0.186[a] 0.304[c] | | p = 0.436[b] 0.236[c] | | |
| **Gender n (%)** | | | | | | | | | | |
| **Male** | 16 (44.4) | 20 (55.6) | 17 (47.2) | 10 (27.8) | 9 (25) | 17 (47.2) | 19 (52.8) | 17 (47.2) | 13 (36.1) | 6 (16.7) |
| **Female** | 4 (25) | 12 (75) | 4 (25) | 3 (18.8) | 9 (56.3) | 7 (43.8) | 9 (56.3) | 7 (43.8) | 5 (31.3) | 4 (25) |
| | p = 0.183[a] 0.184[c] | | p = 0.089[a] 0.305[c] | | | p = 0.817[a] 0.032[c] | | p = 0.777[a] 0.099[c] | | |
| **Ethnicity n (%)** | | | | | | | | | | |
| **Sindhi** | 7 (31.8) | 15 (68.2) | 8 (36.4) | 6 (27.3) | 8 (36.4) | 12 (54.5) | 10 (45.5) | 12 (54.5) | 6 (27.3) | 4 (18.2) |
| **Pathan** | 4 (30.8) | 9 (69.2) | 4 (30.8) | 4 (30.8) | 5 (38.5) | 5 (38.5) | 8 (61.5) | 5 (38.5) | 5 (38.5) | 3 (23.1) |
| **Punjabi** | 3 (60) | 2 (40) | 3 (60) | 1 (20) | 1 (20) | 4 (80) | 1 (20) | 4 (80) | 1 (20) | 0 |
| **Urdu speaking** | 5 (62.5) | 3 (37.5) | 5 (62.5) | 3 (37.5) | 3 (37.5) | 2 (25) | 6 (75) | 2 (25) | 2 (25) | 3 (37.5) |
| **Hindu** | 1 (25) | 3 (75) | 1 (25) | 2 (50) | 1 (25) | 1 (25) | 3 (75) | 1 (25) | 3 (75) | 0 |
| | p = 0.448[b] | | p = 0.678[b] | | | p = 0.284[b] | | p = 0.364[b] | | |

| | | | | |
|---|---|---|---|---|
| | 0.276[c] | 0.237[c] | 0.322[c] | 0.290[c] |

[a] Chi square test applied
[b] Fishers exact test applied
[c] Cramer's V value
