# Supplementary material for: Integrated Genomic Analysis Reveals the Synergistic Role of PNPLA3 and ABCC8 Variants in Diabetic MASLD in Pakistan
Source: Med Sci (Basel). 2025 Sep 5;13(3):178. doi: 10.3390/medsci13030178 (PMC12452525; doi:10.3390/medsci13030178)
Supplement: Supplementary file 1 [file medsci-13-00178-s001.zip › Table S3.pdf]

Table S3: Multiple linear regression of lab parameters with rs738409 and rs146378237

Model	Unstandardized β Coefficient (95% CI)	Standard Error	t	p-value
**Age**				
**rs738409**				
**CC**	Ref.			
**GC**	-7.441 (-17.630 to 2.748)	5.065	-1.469	0.148
**GG**	-1.268 (-12.398 to 9.862)	5.533	-0.229	0.820
**rs146378237**				
**CC**	Ref.			
**TC**	0.564 (-9.170 to 10.298)	4.838	0.117	0.908
**TT**	3.334 (-9.457 to 16.125)	6.358	0.524	0.602
**Weight**				
**rs738409**				
**CC**	Ref.			
**GC**	12.732 (0.192 ton25.272)	6.234	2.042	0.047*
**GG**	8.949 (-4.75 to 22.648)	6.809	1.314	0.195
**rs146378237**				
**CC**	Ref.			
**TC**	-9.806 (-21.786 to 2.173)	5.955	-1.647	0.106
**TT**	-13.395 (-29.137 to 2.347)	7.825	-1.712	0.094
**Height**				
**rs738409**				
**CC**	Ref.			
**GC**	4.252 (-3.121 to 11.624)	3.665	1.160	0.252
**GG**	-1.299 (-9.352 to 6.755)	4.003	-0.324	0.747
**rs146378237**				
**CC**	Ref.			
**TC**	0.712 (-6.331 to 7.755)	3.501	0.203	0.840
**TT**	-2.423 (-11.678 to 6.832)	4.600	-0.527	0.601
**BMI**				
**rs738409**				
**CC**	Ref.			
**GC**	2.638 (-2.164 to 7.44)	2.387	1.105	0.275
**GG**	4.307 (-0.938 to 9.553)	2.607	1.652	0.105
**rs146378237**				
**CC**	Ref.			
**TC**	-4.018 (-8.605 to 0.569)	2.280	-1.762	0.085
**TT**	-4.879 (-10.907 to 1.149)	2.2996	-1.628	0.110
**ALT**				
**rs738409**				
**CC**	Ref.			

**GC**	10.490 (-4.988 to 25.969)	7.694	1.363	0.179
**GG**	26.994 (10.036 tom43.852)	8.405	3.206	0.002*
**rs146378237**				
**CC**	Ref.			
**TC**	6.674 (-8.112 to 21.460)	7.350	0.908	0.369
**TT**	6.531 (-12.9 to 25.961)	9.659	0.676	0.502
	**AST**			
**rs738409**				
**CC**	Ref.			
**GC**	9.839 (-5.413 to 25.090)	7.581	1.298	0.201
**GG**	6.412 (-10.248 to 23.072)	8.281	0.774	0.443
**rs146378237**				
**CC**	Ref.			
**TC**	-0.927 (-15.496 to 13.642)	7.242	-0.128	0.899
**TT**	0.956 (-18.190 to 20.101)	9.517	0.100	0.920
	**GGT**			
**rs738409**				
**CC**	Ref.			
**GC**	7.062 (-53.534 to 67.657)	30.121	0.234	0.816
**GG**	63.932 (-2.260 to 130.123)	32.903	1.943	0.058
**rs146378237**				
**CC**	Ref.			
**TC**	35.061 (-22.824 to 92.947)	28.774	1.219	0.229
**TT**	9.333 (-66.733 to 85.400)	37.811	0.247	0.806
	**ALP**			
**rs738409**				
**CC**	Ref.			
**GC**	16.964 (-23.381 to 57.308)	20.054	0.846	0.402
**GG**	41.284 (-2.787 to 85.354)	21.906	1.885	0.066
**rs146378237**				
**CC**	Ref.			
**TC**	6.845 (-31.695 to 45.385)	19.157	0.357	0.722
**TT**	-24.075 (-74.720 to 26.569)	25.175	-0.956	0.344
	**Total Bilirubin**			
**rs738409**				
**CC**	Ref.			
**GC**	-0.286 (-0.764 to 0.192)	0.238	-1.203	0.235
**GG**	-0.042 (-0.565 to 0.480)	0.260	-0.162	0.872
**rs146378237**				
**CC**	Ref.			
**TC**	0.171(-0.286 to 0.628)	0.227	0.753	0.455
**TT**	0.043 (-0.557 to 0.644)	0.298	0.144	0.886

**Direct Bilirubin**				
**rs738409**				
**CC**	Ref.			
**GC**	0.055 (-0.91 to 0.202)	0.073	0.762	0.450
**GG**	0.035 (-0.124 to 0.195)	0.079	0.446	0.657
**rs146378237**				
**CC**	Ref.			
**TC**	0.088 (-0.052 to 0.227)	0.069	1.263	0.213
**TT**	0.108 (-0.075 to 0.292)	0.091	1.185	0.242
**Albumin**				
**rs738409**				
**CC**	Ref.			
**GC**	0.016 (-0.594 to 0.626)	0.303	0.052	0.959
**GG**	0.193 (-0.473 to 0.859)	0.331	0.583	0.563
**rs146378237**				
**CC**	Ref.			
**TC**	-0.046 (-0.628 to 0.537)	0.290	-0.158	0.875
**TT**	-0.159 (-0.925 to 0.606)	0.380	-0.418	0.678
**Platelet count**				
**rs738409**				
**CC**	Ref.			
**GC**	18.638 (-56.355 to 93.632)	37.278	0.500	0.619
**GG**	11.369 (-70.550 to 93.288)	40.721	0.279	0.781
**rs146378237**				
**CC**	Ref.			
**TC**	-44.075 (-115.715 to 27.564)	35.611	-1.238	0.222
**TT**	-33.702 (-127.843 to 60.438)	46.795	-0.720	0.475
**HbA1c**				
**rs738409**				
**CC**	Ref.			
**GC**	-0.514 (-1.260 to -.232)	0.371	-1.387	0.172
**GG**	-0.001 (-0.816 to 0.814)	0.405	-0.003	0.997
**rs146378237**				
**CC**	Ref.			
**TC**	-0.008 (-0.721 to 0.704)	0.354	-0.023	0.981
**TT**	0.530 (-0.406 to 1.467)	0.466	1.139	0.260
**FBS**				
**rs738409**				
**CC**	Ref.			
**GC**	-1.972 (-21.796 to 17.853)	9.854	-0.200	0.842
**GG**	-3.946 (-25.601 to 17.709)	10.764	-0.367	0.716
**rs146378237**				
**CC**	Ref.			
**TC**	4.103 (-14.835 to 23.041)	9.414	0.436	0.665
**TT**	16.457 (-8.429 to 41.343)	12.370	1.330	0.190
**Insulin**				
**rs738409**				

**CC**	Ref.			
**GC**	3.400 (-3.484 to 10.285)	0.994	0.994	0.325
**GG**	1.072 (-6.448 to 8.593)	0.287	0.287	0.776
**rs146378237**				
**CC**	Ref.			
**TC**	-1.501 (-8.078 to 5.076)	-0.459	-0.459	0.648
**TT**	-0.656 (-9.298 to 7.987)	-0.153	-0.153	0.879
	**HOMA-IR**			
**rs738409**				
**CC**	Ref.			
**GC**	-0.805 (-2.325 to 0.714)	0.755	-1.066	0.292
**GG**	-0.378 (-2.037 to 1.282)	0.825	-0.458	0.649
**rs146378237**				
**CC**	Ref.			
**TC**	0.159 (-1.293 to 1.610)	0.721	0.220	0.827
**TT**	0.896 (-1.011 to 2.804)	0.948	0.945	0.349
	**Total Cholesterol**			
**rs738409**				
**CC**	Ref.			
**GC**	2.952 (-24.563 to 30.467)	13.677	0.216	0.830
**GG**	12.155 (-17.901 to 42.211)	14.940	0.814	0.420
**rs146378237**				
**CC**	Ref.			
**TC**	-3.524 (-29.808 to 22.760)	13.065	-0.270	0.789
**TT**	17.365 (-17.174 to 51.905)	17.169	1.011	0.317
	**Triglycerides**			
**rs738409**				
**CC**	Ref.			
**GC**	-20.882 (-70.490 to 28.726)	24.659	-0.847	0.401
**GG**	1.066 (-53.123 to 55.255)	26.937	0.040	0.969
**rs146378237**				
**CC**	Ref.			
**TC**	-35.742 (-83.131 to 11.647)	23.556	-1.517	0.136
**TT**	-32.171 (-94.445 to 30.102)	30.955	-1.039	0.304
	**HDL**			
**rs738409**				
**CC**	Ref.			
**GC**	2.732 (-4.963 to 10.427)	3.825	0.714	0.479
**GG**	1.873 (-9.533 to 10.278)	4.178	0.448	0.656
**rs146378237**				
**CC**	Ref.			
**TC**	-9.279 (-16.630 to -1.928)	3.654	-2.539	0.014*
**TT**	-4.141 (-13.800 to 5.519)	4.802	-0.862	0.393
	**LDL**			
**rs738409**				

**CC**	Ref.			
**GC**	-10.338 (-35.149 to 14.472)	12.333	-0.838	0.406
**GG**	-0.874 (-27.975 to 26.228)	13.472	-0.065	0.949
**rs146378237**				
**CC**	Ref.			
**TC**	-11.837 (-35.537 to 11.864)	11.781	-1.005	0.320
**TT**	20.917 (-10.227 to 52.061)	15.481	1.351	0.183
		**Steatosis score**		
**rs738409**				
**CC**	Ref.			
**GC**	-5.286 (-27.145 to 16.574)	10.866	-0.486	0.629
**GG**	8.769 (-15.110 to 32.647)	11.870	0.739	0.464
**rs146378237**				
**CC**	Ref.			
**TC**	3.052 (-17.830 to 23.934)	10.380	0.294	0.770
**TT**	-1.610 (-29.051 to 25.831)	13.640	-0.118	0.907
		**Fibrosis score**		
**rs738409**				
**CC**	Ref.			
**GC**	2.051(-1.236 to 5.338)		1.255	0.216
**GG**	3.648 (0.058 to 7.238)	1.634	2.044	0.047*
		1.785		
**rs146378237**				
**CC**	Ref.			
**TC**	1.319 (-1.821 to 4.459)	1.561	0.845	0.401
**TT**	3.789 (-0.337 to 7.915)	2.051	1.847	0.071

Multiple linear regression test applied
*Significant at $p < 0.05$

BMI: body mass index; ALT: alanine aminotransferase; AST: aspartate transaminase; ALP: alkaline phosphatase; GGT: gamma-glutamyl transpeptidas; HDL: high density lipoprotein; LDL: low density lipoprotein; FBS: fasting blood sugar; HOMA-IR: homeostatic model for insulin resistance; HbA1c: glycated hemoglobin; APRI: AST to platelet ratio index; NFS: NAFLD fibrosis score; Fib-4: fibrosis-4; CI: Confidence interval
