# Supplementary material for: Integrated Genomic Analysis Reveals the Synergistic Role of PNPLA3 and ABCC8 Variants in Diabetic MASLD in Pakistan
Source: Med Sci (Basel). 2025 Sep 5;13(3):178. doi: 10.3390/medsci13030178 (PMC12452525; doi:10.3390/medsci13030178)
Supplement: Supplementary file 1 [file Figure S2.pdf › Figure S2.pdf]

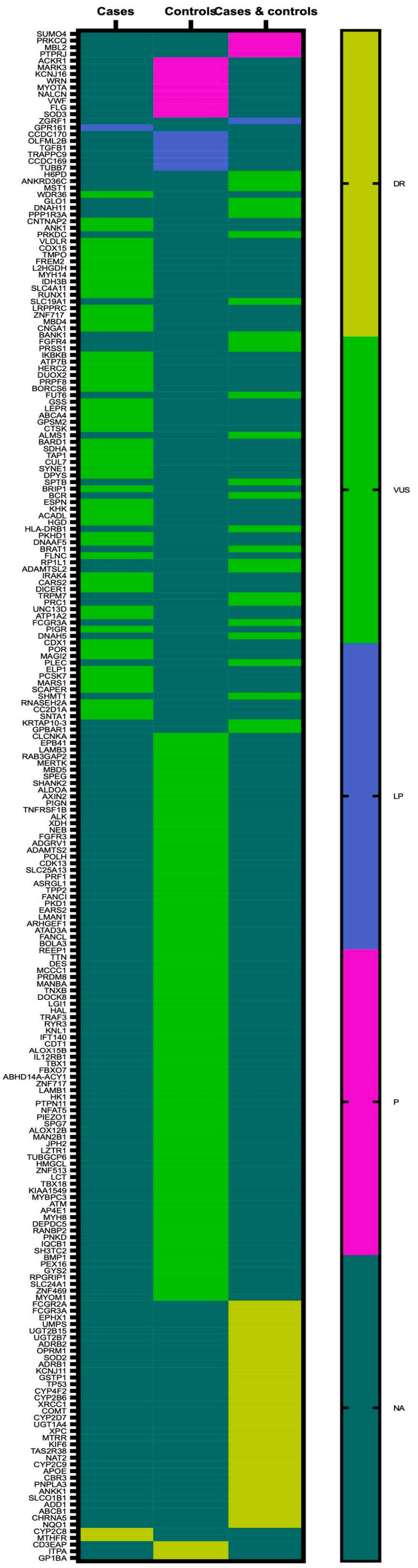


**Figure S2: Heatmap displaying variants identified by CLINVAR analysis**

**Cases:** P =0; LP = 1, VUS = 57, DR = 2; **Controls:** P = 9, LP = 6, VUS = 85, DR = 3; **Cases & Controls:** P = 4, LP = 1, VUS = 27, DR =34

(P = pathogenic; LP= likely pathogenic; VUS=variant of uncertain significance; DR= drug response; NA=not applicable)
